# Targeting Calcium Signalling in Malignant Mesothelioma †

**DOI:** 10.3390/cancers11121839

**Published:** 2019-11-21

**Authors:** Simona Martinotti, Mauro Patrone, Francesco Moccia, Elia Ranzato

**Affiliations:** 1 DiSIT-Dipartimento di Scienze e Innovazione Tecnologica, University of Piemonte Orientale, Viale Teresa Michel 11, 15121 Alessandria, Italy; mauro.patrone@uniupo.it (M.P.); elia.ranzato@uniupo.it (E.R.); 2 Dipartimento di Scienze e Innovazione Tecnologica, University of Piemonte Orientale, Piazza Sant’Eusebio 5, 13100 Vercelli, Italy; 3 Department of Biology and Biotechnology “L. Spallanzani”, University of Pavia, 27100 Pavia, Italy; francesco.moccia@unipv.it

**Keywords:** Ca^2+^ channel, Ca^2+^ toolkit, Cancer, Mesothelioma, Signalling pathway

## Abstract

Calcium ions (Ca^2+^) are central in cancer development and growth, serving as a major signaling system determining the cell’s fate. Therefore, the investigation of the functional roles of ion channels in cancer development may identify novel approaches for determining tumor prognosis. Malignant mesothelioma is an aggressive cancer that develops from the serosal surface of the body, strictly related to asbestos exposure. The treatment of malignant mesothelioma is complex and the survival outcomes, rather than the overall survival data are, to date, disappointedly daunting. Nevertheless, conventional chemotherapy is almost ineffective. The alteration in the expression and/or activity of Ca^2+^ permeable ion channels seems to be characteristic of mesothelioma cells. In this review, we explore the involvement of the Ca^2+^toolkit in this disease. Moreover, the established sensitivity of some Ca^2+^channels to selective pharmacological modulators makes them interesting targets for mesothelioma cancer therapy.

## 1. Introduction

Cancer cells embody characteristics that allow them to survive beyond their normal life span and to proliferate abnormally, presenting defects in the mechanisms underlying cellular growth, migration, and death [1].

Calcium ions (Ca^2+^) play a pivotal role in these processes, acting as principal signalling agent and the expression of Ca^2+^ channel transcripts have been highlighted as a potential biomarker in the growing number of cancers [2,3]. Furthermore, a increasing number of works have revealed that tumor suppressors and oncogenes regulate Ca^2+^ transport systems, including inositol 1,4,5-trisphosphate (IP_3_) receptors (IP_3_Rs) [4]. For that reason, the investigation of the functional roles of ion channels in cancer development may identify novel approaches for determining tumour prognosis [5] and the discovery that alteration in key Ca^2+^-transport molecules could underlie pathological changes, might provide promising targets for treatment of tumours. 

Biomarkers are considered as a cost-effective means of controlling neoplasms, and in the last 30 years, many investigations for a mesothelioma biomarker has taken place [6]. Although Ca^2+^ transport and Ca^2+^ expression is still a novel research area in oncology, and in particular for mesothelioma, prompt development of the field ensures the advancement of improved molecular Ca^2+^ transport-targeting tools for the diagnosis and treatment of mesothelioma patients. 

## 2. Mesothelioma Biology

Malignant mesothelioma is an aggressive tumour rising from the serosal surface of the body. Pleural mesothelioma represents about 80% of mesothelioma diagnoses. Uncommonly, other serosal membranes of the human body can be affected such as the peritoneum (peritoneal mesothelioma), pericardium (pericardial mesothelioma), and tunica vaginalis (tunica vaginalis mesothelioma) [7].

Mesothelioma is normally linked to prior asbestos exposure (job-related or, less frequently, domestic or environmental exposure). Malignant pleural mesothelioma is a sporadic tumour, however the annual rate differs from ten cases per million people (in the USA) to 29 cases per million people (in the UK and in Australia), in agreement to national registries in some countries. The mesothelioma frequency appears, for some countries such as USA, to have reached a plateau (around 3200 per year). Conversely, for several European countries the incidence peak is not expected before the 2020s, due to the long latency period between asbestos exposure and diagnosis (up to 30–50 years), and the widespread asbestos use until the 1970s in most high-income countries [8]. Moreover, asbestos still not being banned worldwide, and its mining and usage is continuing in several countries (e.g., India, China, Russia, and Kazakhstan). According to 2013 WHO predictions, this continuous use of asbestos could be responsible for an epidemic of asbestos-related illnesses in the next decades [8].

Subsequent to inhalation, asbestos fibres can reach the lung edge and the pleura producing a local chronic inflammation and other oncogenic effects [9]. In recent years, some molecular alterations and new targets were identified in mesothelioma [10], such as growth factors and angiogenic pathways, regulators of cell-cycle and apoptosis (i.e., NF-κB pathway), and epigenetic modulators (acetylation/deacetylation of DNA) [11].

The management of mesothelioma is difficult and the outcomes can be disheartening. Cancer cells are resistant to treatments and patients are typically identified at an advanced disease stage, due to the late and generic symptoms [11]. The use of radiotherapy is rare and is usually reserved for pain relief induced by the infiltration of the chest wall [8]. Most international guidelines recommend that surgery should be part of a multimodal treatment regimen (i.e., surgery combined with chemotherapy, radiotherapy, or both). First-line chemotherapy normally combines pemetrexed and cisplatin or pemetrexed and carboplatin. Raltitrexed represents, as first-line chemotherapy, a valid alternative to pemetrexed, associated to cisplatin [8]. No therapy has been yet approved or formally endorsed beyond first-line treatment for malignant pleural mesothelioma, except to consider a repeat course of chemotherapy with pemetrexed [8].

Based on these data, malignant mesothelioma is a sporadic disease with scarce therapeutic choices. Moreover, there are several unresolved issues, such as the lack of predictive biomarkers and adequate tools for response evaluation. Therefore, it is mandatory to improve our knowledge of the mesothelioma biology.

## 3. The Intracellular Ca^2+^ Toolkit

The intracellular Ca^2+^ concentration [Ca^2+^]_i_ in unstimulated cells is around 100 nM, whereas the concentration is higher in the extracellular space and in the intracellular Ca^2+^ stores, ranging from 1 to 2 mM. Although the [Ca^2+^]_i_ is maintained very low, small variations, due to intracellular release from endogenous stores, or to extracellular Ca^2+^ entry may induce local or global Ca^2+^ signals, thereby activating specific Ca^2+^-dependent downstream pathways [12]. 

Intracellular Ca^2+^, which represents one of the most widespread second messengers, plays a crucial role in the control of cellular processes, and this plethora of Ca^2+^ activities is primarily induced through spatial localization, magnitude, and temporal variations in cytosolic Ca^2+^ concentrations—local spikes of Ca^2+^, transient waves of intracellular Ca^2+^, and global fluttering, ranging from rapid events (neurosecretion and muscle contraction), to slower responses (cell division and differentiation, and the apoptotic pathway) [13].

**Figure 1:**
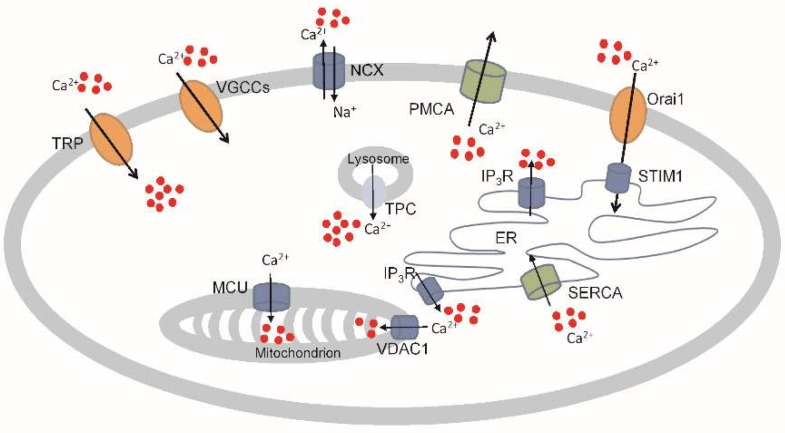
Examples of Ca^2+^-permeable channels, pumps, and plasma membrane and intracellular organelles exchangers. Ca^2+^ entry from the extracellular space is mediated by plasma membrane channels including Transient Receptor Potential channel (TRP ) and Voltage-Gated Ca^2+^ Channel (VGCC), and the plasma membrane transporters such as NCX (Na^+^/Ca^2+^ exchanger) and PMCA (Plasma Membrane Ca^2+^-ATPase) allow for the removal of Ca^2+^ from the cytoplasm. Upon the depletion of internal Ca^2+^ stores, a particular way of Ca^2+^ entry is through the store-operated calcium entry (SOCE) channels, Orai1 (Ca^2+^ release-activated Ca^2+^ modulator 1), and STIM (Stromal Interaction Molecule). The movement of Ca^2+^ toward intracellular stores and the cytoplasm occurs by intracellular calcium channels and transporters as well as endoplasmic reticulum channels, IP_3_R (Inositole 3 Phosphate Receptor) and RyR (Ryanodine Receptor), and transporters, SERCA (Sarco/Endoplasmic Reticulum Ca^2+^-ATPase); mitochondrial transporter, MCU (Mitochondrial Ca^2+^ Uniporter) and channels, VDAC1 (Voltage Dependent Anion Channel 1); and lysosomal channel, TPC (Two-porte channel).

A network of different Ca^2+^ channels and transporters, referred to as the “Ca^2+^ signalling tookit”, regulates the intracellular homeostasis of Ca^2+^ (see Figure 1). Here, however, we will not focus our interest on normal trans-membrane transport mechanisms, but instead, we will explore the connections between the altered expression of specific Ca^2+^ channels/pumps and cancer development, with particular attention to malignant mesothelioma [14].

## 4. Remodelling of the Ca^2+^ Toolkit in Cancer 

Cancer relapse with treatment resistance underlines the strong necessity to recognise new molecular targets for the creation of different therapies. 

In recent times, the role of ion channels in influencing malignant cancer cell behaviour has been discovered and much has been obtained from brain cancer. In fact, the first direct suggestions that ion channels play a crucial role in carcinogenesis came in the late 1980s, with studies describing that brain cancer cells show uncommon configurations of ion channel functional expression and that blockade by pharmacological inhibitions of some channels can hinder the growth of tumour cells [15]. Among genes altered during cancer progression, it is expected that those coding for ion channels are present. Several data have been gathered suggesting that the alteration of ion channels can be prominent to the hallmarks of cancer. The remodelling of intracellular Ca^2+^ signals and Ca^2+^ homeostasis is assumed as key events in inducing, or sustaining, malignant behaviours. Definitely, cancer alteration is linked with a Ca^2+^-transporting molecules rearrangement (changes in function and/or expression), which contributes with other signalling pathways. This could result in improved survival (apoptosis evasion), disproportionate cellular growth, cell migration, malignant angiogenesis, and metastatisation [16]. So, due to the increased knowledge of ion pumps and channels involved in neoplasm growth, tumours can be categorised as a channelopathy, or a disease induced by the disturbed function of ion channels, frequently due to channel expression deregulation (transcriptional channelopathy) or other alterations resultant in transformed function [15]. Many examples of the Ca^2+^-toolkit remodelling and Ca^2+^ mishandling are reported and some of these variations have been suggested to be important drivers stimulating and preserving the cancer phenotype [17]. Channels in the plasma membrane allow for the entry of Ca^2+^ into the cytoplasm, alongside the concentration gradient across the plasma membrane. Many Ca^2+^ channels, such as voltage-gated Ca^2+^ channels (Ca_v_ family), can be activated in this Ca^2+^ influx. Members of the low voltage-activated (low threshold of activation) Ca_v_3 subfamily are expressed or overexpressed in different tumour cells [18]. Despite Ca_v_ expression, Ca^2+^ entry into non-excitable cells classically happens by way of non-voltage-gated channels, such as Receptor-Operated Channels (ROC) or Secondary Messenger-Operated Channels (SMOC) linked to GPCR (G-Protein Coupled Receptor) activation—the Orai family and members of the TRP (Transient Receptor Potential) superfamily of channels; Store-Operated Channels (SOC: Orai family and members of TRPC (TRP Canonical) subfamily of channels); ligand-gated channels (P2X purinergic ionotropic receptor families, for instance); and stretch-operated channels (members of TRP channel superfamily). In the literature, one or several of these Ca^2+^-permeable channels present in the plasma membrane have been revealed to be altered in expression and/or activity in many cancer cells, with a pivotal role in some pathophysiological processes leading to the malignant phenotype [19]. Store-Operated Ca^2+^ entry (SOCE) has been described to play a central role in cell proliferation, cycle progression, migration, and metastasis as well as apoptosis evasion [20]. There is an increasing number of studies that support this fundamental role for SOCE. These data underlie the phenotypic alterations of tumour cells that suggest the Stromal Interaction Molecule 1 (STIM1) and the SOC channels as appropriate candidate targets for future prognostic or therapeutic plans [21].

In addition to the plasma membrane Ca^2+^ channels, alterations in Ca^2+^-transporters, such as the Plasma Membrane Calcium ATPase (PMCA) and Na^+^/Ca^2+^ exchanger, have also been suggested to play a pivotal role in Ca^2+^ homeostasis of neoplastic cells [22]. 

Similarly, mutations as well as transformed expression levels of Sarco/Endoplasmic Reticulum Calcium ATPase (SERCA) isoforms have been involved in many tumours, including lung, prostate, and colon cancers [23]. Also, mitochondria can store an important amount of Ca^2+^ within their matrix, 10-fold higher than the homeostatic cytosolic concentration. Ca^2+^ is transported from the ER via specialized regions, called mitochondria-associated membranes (MAMs) [24]. There is a clear connection between MAMs and cancer, derived from observations on promyelocytic leukemia (PML) and Akt, a proto-oncogene frequently upregulated in tumour. The phosphorylation state of IP_3_R is a significant characteristic in defining the amount of Ca^2+^ efflux from the ER to the mitochondria [25]. In the MAMs regions, the presence of the PML pool, is fundamental to allow the transmission of pro-apoptotic Ca^2+^ signals across IP_3_R to the mitochondria, that lead to mitochondrial Ca^2+^ overload and the consequent apoptotic process under stress conditions [26]. Similarly, PTEN can also impact Akt/IP_3_R signalling by localizing at the MAMs, demonstrating again that the ER–mitochondria contact sites are leading locations for the regulation of cell fate in cancer [27].

The Ca^2+^-permeable channels of the plasma membrane are encouraging candidates as targets for pharmacological approaches. Modulators of the Ca^2+^ toolkit have been revealed to decrease or oppose malignant behaviour in different neoplastic cells. The low sensitivity of pharmacological drugs is one of the main limits for clinical utilization. However, hindering or controlling some of the Ca^2+^ channels, which are strongly expressed in tumour cells, and show a circumscribed tissue distribution, could avoid general concerns. The study of the Ca^2+^ toolkit and homeostasis in neoplastic cells is a slightly new but auspicious research area.

## 5. Ca^2+^ Signalling and Mesothelioma

Until now, few data are available for the role of Ca^2+^ in mesothelioma (see Table 1). 

### 5.1. Calcium Activated Potassium Channels

Since mesothelioma is characterized by mutations in many genes, a genome-wide gene expression profile, obtained by microarray and next generation sequencing, allows for the identification of precise expression profiles. Using such an approach, Cheng et al. [28] described the contribution of microRNAs in the regulation of mesothelioma growth. In particular, they showed the minor expression of miR-17-5p and miR-20a-5p in epithelioid histology. Among the predicted targets of these differentially expressed microRNA families, they identified K_Ca_1.1, a Ca^2+^-activated potassium channel subunit alpha 1 encoded by the KCNMA1 gene, as a target of miR-17-5p. K_Ca_1.1 was expressed at higher levels in mesothelioma cells compared to the (normal) mesothelial line MeT-5A, and was highly expressed in the patient tumour specimen compared to the normal mesothelium [28]. Potassium ion channels such as K_Ca_1.1 are the topic of growing interest in oncology due to their roles in cellular processes such as cell growth, cell migration, cell adhesion, new vessel formation, and metastasis [37]. In mesothelioma cells, KCNMA1 knockdown induces a sustained increase in the basal [Ca^2+^]_i_. As KCNMA1 contributes to the resting membrane potential, this effect seems to be due to an incomplete membrane depolarization and greater activity of Ca^2+^ channels [38]. Additional studies are requested to appreciate accurately how KCNMA1 activity loss and resultant variations in Ca^2+^ flux decreases migration and invasion without affecting apoptosis. Some studies are suggesting that the potassium channels, such as Kv10.1, Kv11.1, K_Ca_1.1, and K_Ca_3.1, may have central parts in tumour cell invasion and metastatic processes [39], and targeting these channels is a potential therapeutic option for mesothelioma treatment. 

### 5.2. T-Type Calcium Channel 

An increasing body of data have suggested the potential for voltage-activated Ca^2+^ channels, in particular T-type, in the regulation of cancer proliferation and evolution.

Molecular biology works divided these Ca^2+^ channels in three main subfamilies called Ca_v_1, Ca_v_2, and Ca_v_3. Ca_v_3 channels Ca_v_3.1 (α1G), Ca_v_3.2 (α1H), and Ca_v_3.3 (α1I) are low voltage-activated, dihydropyridine-sensitive, T-type or ‘transient currents’ indicating their kinetics of activation and inactivation [40]. At low voltages, T-type Ca^2+^ channels generate the so-called ‘window current’ at membrane potential-appropriate values, resulting in a sustained inward Ca^2+^ current. This regulation of Ca^2+^ homeostasis permits T-type Ca^2+^ channels to control cell growth and differentiation. Therefore, the loss of T-type Ca^2+^ channel control may lead to abnormal cell proliferation and cancer progression [41].

Recently we have demonstrated that T-type Ca^2+^ channels are highly present in mesothelioma patient tissues compared to in normal mesothelial tissue [29]. In particular, we found the overexpression of Ca_v_3.2 α1H protein encoded by the CACNA1H gene. Then, we elucidated the role of T-type Ca^2+^ channels as the transducing factor between the epigallo-catechin-3-gallte (EGCG)-induced extracellular H_2_O_2_ release and the intracellular harmful effects observed in mesothelioma cells. The demonstration of this mechanism was suggested by the preventive action of mibefradil and Ni^2+^ on Ca^2+^ homeostasis disturbance induced by EGCG. Hereafter, to obtain a more direct evaluation, we have targeted the Ca_v_3.2 isoform. The higher expression of this channel in mesothelioma cell lines, with respect to normal mesothelium, elucidates the selective toxicity mechanism of EGCG to mesothelioma. Therefore, with the reconstruction of the selective mechanism of EGCG toxicity to mesothelioma cells, we have pointed out T-type Ca^2+^ channels as a potential novel therapeutic target for this neoplasia.

Moreover, as a confirmation of the role of T-type channels, another work [30] has demonstrated that a polyphenol, resveratrol, induced [Ca^2+^]_i_ dysregulation in mesothelioma cells by stimulation of T-type Ca^2+^ channels.

Recent literature have discussed the idea of pharmacological inhibition or RNAi-mediated downregulation of T-type Ca^2+^ channels as a way to inhibit cancer cell growth and increase cancer cell death [42]. In addition to a single agent activity, the results validate that T-type Ca^2+^ channel blockers improve the anticancer properties of conventional radio- and chemotherapy [43]. Consequently, T-type Ca^2+^ channels could be attractive molecular targets for the improvement of mesothelioma treatment, considering the actual inefficacy of classic therapy.

### 5.3. Calcium Binding Protein

Calretinin, a 29-kD calcium-binding protein, initially detected in the central nervous system, is expressed in benign mesothelial cells and mesothelioma. Staining is both cytoplasmatic and nuclear, with nuclear staining required for the epithelioid and mixed (biphasic) mesothelioma identification, to distinguish from metastatic breast or lung cancer [31]. Calretinin is suggested to have a pivotal role in the initiation process of mesotheliomagenesis [32], being essential for cell growth and survival.

Blum and co-workers showed that mouse mesothelial cell proliferation and migration was augmented or decreased, respectively, by the overexpression or absence of calretinin, suggesting this protein as a possible target [33].

### 5.4. BRCA1-Associated Protein 1 (BAP1)

BRCA1-associated protein 1 (BAP1) is a deubiquitinase enzyme, a member of the ubiquitin carboxy (C)-terminal hydrolase family, involved in the regulation of some cellular pathways, ranging from cell cycle regulation, to cell death, metabolism, and the DNA damage response [44]. Mutations and deletions determining BAP1 loss have been described in several tumors including lung, breast, melanoma, and mesothelioma [45,46]. People with one inactive BAP1 allele (BAP1 tumour predisposition syndrome) have a significantly higher predisposition to cancer, and somatic BAP1 point mutations were present in up to 60% of sporadic mesothelioma [34].

Bononi et al. [47] described that BAP1 is present in the endoplasmic reticulum (ER) where it deubiquitylates, stabilizes, and modulates the activity of type 3 IP_3_R (IP_3_R3). The IP_3_R3 on the ER membrane regulates the Ca^2+^ flux release from the ER to the mitochondria. In cells that carry the BAP1 mutation, its resultant low level determines a reduction in the release of Ca^2+^ from the ER leading to low mitochondrial Ca^2+^ concentration, which in turn reduces the capacity of BAP1+/- cells to _complete_ the apoptotic pathway. Furthermore, mesothelioma cells lacking of BAP1, for acquired or inherited mutations, are resistant to gemcitabine-induced apoptosis [48]. A novel mechanism for BAP1 has been proposed in neuroblastoma [49] demonstrating that the association between BAP1 and 14-3-3 protein can induce the release of the apoptotic inducer protein Bax from 14-3-3 and so promoting cell death through the intrinsic apoptotic pathway. Therefore, other observations are strongly required to assess the role of BAP1 on mesothelioma biology and with particular attention to drugs used for this cancer such as pemetrexed and platinum-based treatments, as well as to avoid second line treatment with compounds not effective in BAP1 mutant patients [48].

### 5.5. ER-Mitochondrial Ca^2+^ Handling

Some authors [35] have already suggested that mineral asbestos, considered as the main cause of mesothelioma onset, can induce an ER-stress response, moreover altering ER Ca^2+^ release. 

The earliest works on asbestos exposure effects on intracellular Ca^2+^ suggested that the exposure of polymorphonuclear leucocytes (PMNs) to asbestos fibers increases cell membrane permeability, measured as the release of lactate dehydrogenase, not observed in the absence of extracellular Ca^2+^ [50]. Similar results have been observed for alveolar macrophages [51]. However, this latter study suggests also that asbestos fibers can open Ca^2+^ channels on the macrophage membrane, permitting extracellular Ca^2+^ to enter and contribute to the increase in cytosolic Ca^2+^ levels.

Then, Kamp et al. [52] explored how asbestos fibers can initiate the ER stress response to activate the mitochondria-regulated cell apoptotic pathway, demonstrating that, in alveolar epithelial cells, asbestos induces an ER stress response that determines Ca^2+^ release from the ER, playing a crucial role in augmenting mitochondria-regulated apoptosis, an important early event in the pathogenesis of pulmonary fibrosis. This finding suggested the presence of an important cross-talk between the mitochondria and ER in alveolar epithelial cells exposed to oxidant-induced toxicity, such as asbestos fibres.

The ER and mitochondria are physically and functionally interconnected organelles, which modulate mitochondrial metabolism, intracellular Ca^2+^ concentration, and complex cell survival/death signals [53]. B-cell lymphoma 2 (Bcl-2) family members have a significant role in regulating ER/mitochondrial cross talk. The transient ER Ca^2+^ release can boost pro-survival signalling, while intrinsic apoptotic stimuli necessitates the sustained ER Ca^2+^ release along with mitochondrial Bax/Bak binding. Bax and Bak are normally requested to preserve homeostatic levels of ER Ca^2+^ required for regulating intrinsic apoptosis, while Bax/Bak mitochondrial localization is enough for activating BH3-only induced cell death [54]. ER stress can prompt intrinsic apoptosis by activating the unfolded protein response (UPR) [55]. These and other observations suggest the important role for the cross talk between the mitochondria and ER in modulating intrinsic apoptosis. [52]

Given the role of Ca^2+^ in many key cellular processes, it will play a possible role in the functional responses to asbestos, including the carcinogenic process. Mutation and alterations of many key genes in cellular signalling, which are responsible for the control of both apoptosis and Ca^2+^ handling, have been recognised in mesothelioma histologic samples and are considered responsible for the onset/progression of mesothelioma [56,57].

Patergnani and colleagues [36] have revealed a critical role for Ca^2+^ ions in the control of apoptosis in mesothelioma cells. By comparing mesothelioma cells with normal mesothelial cells, various signalling and apoptotic parameters were evaluated. Increased expression of AKT, BCL-2, and BCL2L1 was found, whereas the expression of PML, a suppressor of oncogenesis, was reduced. They also found a reduced level of the Mitochondrial Calcium Uniporter (MCU), highlighting that the alteration of mitochondrial Ca^2+^ uptake is crucial for the unresponsiveness of mesothelioma cells to apoptotic stimuli, and silencing MCU by RNAi or NaV (sodium orthovanadate) treatment to restore the Ca^2+^ homeostasis and mesothelioma sensitivity to apoptotic stimuli [29,30,34,40,41,42,43,44,45,46,47,48,58].

## 6. Conclusions

It is becoming clear that Ca^2+^ channels/transporters/pumps play a pivotal role in a huge number of neoplasms. In fact, deregulated Ca^2+^ homeostasis could act like a “driver”, rather than a “passenger” in carcinogenesis or tumorigenesis [49]. This novel research field has already obtained important contributions in the identification of potential chemotherapeutic agents for a certain number of neoplasms, with a few even moved to clinical trials [49]. Since some of these Ca^2+^ channels have a role in physiological cell activities, one challenge in drug discovery, affecting these Ca^2+^ signalling proteins, is to recognise their cancer specific roles. 

Despite huge efforts made in recent years, the development of therapies for mesothelioma continue to be challenging and the outcome for patients remains disappointedly poor. There is, therefore, an unmet need to identify more reliable diagnostic and/or prognostic markers and to highlight novel molecular targets to bolster chemotherapy in mesothelioma patients. Chemotherapy is the main strategy for cancer management, but a major challenge limiting its realisation is the occurrence of an intrinsic or acquired drug resistance. 

The important role of Ca^2+^ signalling in growth, cell death, invasion, and metastatic process, offers many chances for targeting altered Ca^2+^ signalling during tumorigenesis course. Moreover, some authors are arguing that the regulation of gene expression in drug resistant cells via [Ca^2+^]_i_ may play an important role in acquired drug resistance [59]. Models comparing the Ca^2+^ signal between neoplastic and non-neoplastic phenotypes, coupled with the use of high throughput and/or advanced Ca^2+^ imaging approaches, might further support the translation of deregulated Ca^2+^ signalling assessment into the development of targeted tumour therapy [59].

Moreover, the changes in the expression and/or activity of Ca^2+^ permeable ion channels seem to be a distinguishing sign of mesothelioma cells. The clearly recognised sensitivity of some Ca^2+^ channels to selective pharmacological modulators makes them attractive targets for mesothelioma management. Additional studies are essential to explain the finest therapeutic strategy for mesothelioma as well as the involvement of the Ca^2+^ toolkit in this disease. 

## Figures and Tables

**Table 1:** Altered Ca^2+^ toolkit in malignant mesothelioma.

Major Effects	Ref.
Overexpression of K_ca_1.1 channel	[28]
T-type Ca^2+^ channel upregulation	[29]
[Ca^2+^]_i_ dysregulation resveratrol-induced	[30]
Calretinin	[31,32,33]
BAP1 regulates IP_3_R3-mediated Ca^2+^ flux	[34]
Mineral asbestos can induce ER-stress response	[35]
Critical role for Ca^2+^ in the control of apoptosis	[36]
